# COVID-19 vaccine acceptance and its drivers among Pakistani population

**DOI:** 10.12669/pjms.39.2.6051

**Published:** 2023

**Authors:** Khalid Rehman, Nauman Arif, Muhammad Jawad, Ali Muhammad

**Affiliations:** 1 Khalid Rehman, PhD, MPH, MBBS, Institute of Public Health & Social Sciences, Khyber Medical University, Peshawar, Pakistan; 2 Nauman Arif, Pharm-D, MSc, Institute of Public Health & Social Sciences, Khyber Medical University, Peshawar, Pakistan; 3 Muhammad Jawad, MD, MCPS, MPH, Institute of Public Health & Social Sciences, Khyber Medical University, Peshawar, Pakistan; 4 Ali Muhammad, BS, MPH, Institute of Public Health & Social Sciences, Khyber Medical University, Peshawar, Pakistan

**Keywords:** COVID-19, Vaccination, Acceptance, Hesitancy

## Abstract

**Background & Objective:**

COVID-19 vaccine has become available within a record time but mere availability will not control the pandemic. High vaccine acceptance is required. The objective was to determine COVID-19 vaccine acceptance and its associated factors among Pakistani population.

**Methods:**

An online survey using google form, was conducted from January 31^st^ to February 9^th^, 2021 before the start of the mass vaccination in Pakistan. The questionnaire had questions about demographics plus vaccine hesitancy. We received a total of 1156 responses. Data was analyzed using STATA version 14. We employed descriptive statistics and chi square test.

**Result:**

A total of 1156 responses were received. 65% were male and 35% female. Only 6% were uneducated. Thirty percent had tested positive for COVID-19 earlier. Forty-six percent of the respondents would take (acceptance) a vaccine if available. Forty-eight percent and 45% were confident in using USA/UK and Chinese vaccine respectively. Gender and marital status was statistically significantly associated with vaccine acceptance. Concerns about the side effects were 55% while for efficacy it was 69%. Twenty-three percent were concerned about the permissibility of the vaccine on religious grounds.

**Conclusion:**

Gender and marital status was significantly associated with vaccine acceptance. Forty-six percent respondents were willing to take the vaccine. Among the vaccine hesitant group, respondents were worried about the side effects, safety and religious permissibility of vaccine. Policy makers and all the relevant stakeholders should consider low vaccine acceptance as a major bottleneck and should devise strategies to address this major issue in the fight against COVID-19.

## INTRODUCTION

World Health Organization (WHO) declared COVID-19 Pandemic on 11^th^ March 2020.Symptoms of COVID-19 include cough, fever, fatigue, headache, fatigue, shortness of breath, and loss of taste and smell.^1^ About one third of patients are asymptomatic.^2^ Most of the patients develop mild to moderate symptoms, while about 14% have severe symptoms (shortness of breath, hypoxia).^3^ Respiratory droplets are sources of transmission of spread for COVID-19 from an infected person.

### Background of Covid Vaccine:

Previously no vaccine against an infectious disease had developed in such a short time – and before COVID-19 vaccine no vaccine existed against coronavirus infection. Some vaccines were developed against corona viruses but that were used against animal diseases, like feline coronavirus, canine coronavirus, infectious bronchitis virus in birds only.^4^ In 2005 and 2006, the development of vaccines against SARS was the top most priority of different governments and public health authorities, but the effort was not successful.^5, 6^

### COVID 19 Vaccine:

From the very start of COVID-19, international response was activated for the development of COVID 19 vaccine.^7^ Development of Vaccine was accelerated by pharmaceutical industry and governments an`d by mid-2020, billions of dollars were made available by governments, corporations, university research groups and international health organizations, for the development of COVID -19 vaccine.^8^ In March 2020 four vaccine candidates entered human evaluation.^7^

United Kingdom approved Pfizer–BioNTech vaccine in December 2020, and became the first country to approve Pfizer–BioNTech vaccine. Then things got rolling, WHO also gave emergency approvals to Pfizer, Moderna, Astrazeneca and Sinopharm.

### Vaccine hesitancy:

WHO, defines the term vaccine hesitancy as refusal or delay in accepting vaccines despite its availability.^9^ Vaccine hesitancy is context specific and complex, which varies across place, time and vaccines. The factors that influence it are complacency, convenience (access to vaccines), and confidence. Vaccine hesitancy existed since the invention of vaccination. Narrative of anti-vaccination advocates have been changing over time.^10^ A systematic review has shown that vaccine hesitancy exists all over the world with an average of 40% and in EMRO region 48%.^11^

In Pakistan, vaccine refusal rate for vaccine preventable diseases have been high. It is deemed necessary that vaccine hesitancy of the population is understood before a nationwide mass vaccination drive is started.^12^ This study provides important insight on COVID-19 vaccine acceptance and hesitancy, in Pakistan.

## METHODS

We conducted a cross-sectional study inviting general population from all over Pakistan from January 31^st^ to February 9^th^, 2021 via online social media platforms. The target population was people aged ≥18years. The survey was advertised mainly via three social media platforms (Facebook, WhatsApp and Twitter), though was not limited to it. Data was collected using self-reported, structured and validated questionnaire (online Google forms). The questionnaire consisted of two sections. Section-I had informed consent. Once the participant agreed to take part in the study, then they would be taken to the Section-II. The Section-II of the questionnaire had two parts and a total of 16 questions. Part-A, covered demographic and had seven questions. Part-B, was the main questionnaire and had 9 questions. Seven questions had a likert scale and two had Yes and No responses. The questionnaire was translated to Urdu and pilot tested; changes were made based on the pilot. Though it was available online both in English and Urdu. The questions and response options were structured in a way that the participant would only move forward once a response is selected, hence avoiding any incomplete forms. The survey was open for response between 31^st^ January to 9^th^ February. The identity of people participating in survey was kept confidential.

### Data Analysis:

Data were extracted from the online Google forms, transferred and analyzed by using STATA Version 14. All the categorical variables (gender, profession, vaccine acceptance etc.) were described as frequencies and percentages. Group-testing was performed using chi square test with p ≤ 0.05 considered significance.

### Institutional Review Board Approval:

The study was conducted according to the guidelines of the Declaration of Helsinki, and approved by the ethics committee of Khyber Medical University, Peshawar. The reference number is DIR/KMU-EB/CV/000875/DR.

## RESULTS

A total of 1156 responses were received in the ten days period. The respondents belong to all provinces and regions of Pakistan but the majority of the participants were from the province of Khyber Pakhtunkhwa (KPK) 647 (56%). We had 223 (19.3%), 78 (6.7%), 48 (4.2%) 71 (6.1%) participants from Punjab, Sindh, Baluchistan and Federal area respectively. Majority of the respondents (n=981,80.5 %), were less than 40 years of age. Amongst the total, male participants were 749 (64.8%) while female participants were 407 (35.2%). More than two thirds of the participants were highly educated, including bachelor, master and doctoral level education n=404 (34.9%), n=380 (32.9%), 49 (4.2%) respectively. Respondent’s further details on the characteristics are provided in Table-I.

**Table-I:** Descriptive analysis for categorical variables (n=1156)

Characteristics	Variable	Categories	Frequency	Percentage
Demographic Characteristics	Age	Less than 40	931	80.5%
		40 to 60	194	16.8%
		Greater than 60	31	2.7%
	Gender	Female	407	35.2%
		Male	749	64.8%
	Marital status	Unmarried	496	42.9%
		Married	657	56.8%
		others	3	.3%
	Education status	Not educated	78	6.7%
		Middle	68	5.9%
		Matric	177	15.3%
		Bachelor	404	34.9%
		MA	380	32.9%
		PhD	49	4.2%
	Monthly income	Less than 15,000	113	9.8%
		15001 to 30,000	228	19.7%
		30,001 to 50,000	217	18.8%
		50,001 to 100,000	141	12.2%
		More than 100,000	106	9.2%
		Do not want to disclose	322	27.9%
	Occupation	Health workers	350	30.3%
		Teacher	131	11.3%
		Labourer	39	3.4%
		Unemployed	91	7.9%
		Private job	129	11.2%
		Own business	64	5.5%
		Retired	323	27.9%
		Others	29	2.5%
		Health workers	350	30.3%
	Location	Khyber Pakhtunkhwa	647	56.0%
		Punjab	223	19.3%
		Sindh	78	6.7%
		Balochistan	48	4.2%
		Federal / Islamabad	71	6.1%
		GB	62	5.4%
		AJK	27	2.3%
COVID-19 test and comorbid conditions	Have you ever tested positive for COVID-19	No	808	69.9%
		Yes	348	30.1%
	Do you have an existing chronic disease such as diabetes, hypertension (blood pressure) or chest diseases (asthma etc)	No	985	85.2%
		Yes	171	14.8%
Vaccines Acceptance	If vaccine against COVID-19 is available, would you take it?	No	115	9.9%
		Yes definitely	530	45.8%
		I will wait and see	304	26.3%
		I am not sure	207	17.9%
COVID-19 confidence	Rate your confidence in using UK, USA manufactured (imported) COVID-19 vaccine?	Completely confident	178	15.4%
		Confident	378	32.7%
		Neutral	400	34.6%
		Not Confident	137	11.9%
		Completely not Confident	63	5.4%
	Rate your confidence in using China manufactured COVID-19 vaccine?	Completely confident	118	10.2%
		Confident	401	34.7%
		Neutral	428	37.0%
		Not Confident	144	12.5%
		Completely not Confident	65	5.6%
	Vaccination decreases my chance of getting COVID-19 or its complications?	Strongly agree	166	14.4%
		Agree	561	48.5%
		Neutral	342	29.6%
		Disagree	67	5.8%
		Strongly disagree	20	1.7%
	I am worried that the possible side-effects of COVID-19 vaccination would interfere with my routine activities?	Strongly agree	148	12.8%
		Agree	492	42.6%
		Neutral	358	31.0%
		Disagree	135	11.7%
		Strongly disagree	23	2.0%
	I am concerned about the safety of the COVID-19 vaccination	Strongly agree	374	32.4%
		Agree	428	37.0%
		Neutral	238	20.6%
		Disagree	100	8.7%
		Strongly disagree	16	1.4%
	I am concerned about permissibility of COVID-19 vaccine on religious grounds?	Strongly agree	91	7.9%
		Agree	177	15.3%
		Neutral	459	39.7%
		Disagree	264	22.8%
		Strongly disagree	165	14.3%

Out of 1156 respondents, n=348 (30.1%) had previously tested COVID-19 positive. Total 171 (14.8%) respondents had an existing chronic disease such as cardiovascular disease, diabetes and chronic respiratory tract disease. When asked, if vaccine against COVID-19 is available would you take it, only 530 (45.8%) of the participants said Yes, 115 (9.9%) categorically refused and 304 (26.3%) responded they will wait and see.

Amongst all the respondents, 556 (48.0%) were either confident or completely confident in using USA, UK manufactured COVID-19 vaccine, whereas 519 (44.8%) were either confident or completely confident in using China manufactured COVID-19 vaccine. Out of the total respondents, 727 (62.9%) acknowledged that vaccination decreases their risk of contracting COVID-19 and its complications. Six hundred and forty (55.4%*)* indicated that they are worried about possible side effects of COVID-19 vaccination. Total 802 (69.4%*)* strongly agreed or agreed that they are concerned about the safety of COVID-19 vaccine. When asked about the permissibility of COVID-19 vaccine on religious grounds, 268 (23.2%*)* were concerned. Further descriptive statistics are provided in Table-I.

Table-II shows COVID-19 acceptance and its associated factors, there was no statistically significant association between age group and COVID-19 vaccine acceptance. Gender and marital status were highly statistically significant with *p-value < 0.001* and equal to 0.007, respectively. Education status, monthly income and occupation were statistically significant with *p*-values *<0.001*. “Vaccination decreases my chance of getting COVID-19 or its complications”, worried about the possible side-effects of COVID-19 vaccination, concerned about the safety or religious permissibility had statistically significant association with vaccine acceptance, with *p*-values *<0.001*.

**Table-II:** COVID-19 cross tabulations of covid-19 acceptance and its factors (n=1156)

Characteristics	Variable	Categories	COVID-19 Acceptance				

			No	Yes definitely	I will wait and see	I am not sure	P = Value
Demographic Characteristics	Age	Less than 40	92 (80%)	408 (77%)	259 (85.2%)	172 (83.1%)	0.112
		40 to 60	21 (18.3%)	106 (20%)	38 (12.5%)	29 (14%)	
		Greater than 60	2 (1.7%)	16 (3%)	7 (2.3%)	6 (2.9%)	
	Gender	Female	50 (43.5%)	159 (30%)	100 (32.9%)	98 (47.3%)	<0.001
		Male	65 (56.5%)	371 (70%)	204 (67.1%)	109 (52.7%)	
	Marital status	Unmarried	56 (48.9%)	199 (37.5%)	136 (44.7%)	105 (50.7%)	0.007
		Married	59 (51.3%)	331 (62.5%)	166 (54.6%)	101 (48.8%)	
		others	0 (0%)	0 (0%)	2 (0.7%)	1 (0.5%)	
	Education status	Not educated	8 (7%)	31 (5.8%)	18 (5.9%)	21 (10.1%)	<0.001
		Middle	9 (7.8%)	25 (4.7%)	16 (5.3%)	18 (8.7%)	
		Matric	17 (14.8%)	62 (11.7%)	36 (11.8%)	62 (30%)	
		Bachelor	42 (36.5%)	199 (37.5%)	112 (36.8%)	51 (24.6%)	
		MA	37 (32.2%)	186 (35.1%)	105 (34.5%)	52 (25.1%)	
		PhD	2 (1.7%)	27 (5.1%)	17 (5.6%)	3 (1.4%)	
	Monthly income	Less than 15,000	12 (11.1%)	52 (10%)	30 (10.1%)	19 (9.5%)	<0.001
		15001 to 30,000	36 (33.3%)	105 (20.2%)	49 (16.5%)	38 (18.9%)	
		30,001 to 50,000	16 (14.8%)	96 (18.4%)	76 (25.6%)	29 (14.4%)	
		50,001 to 100,000	11 (10.2%)	66 (12.7%)	43 (14.5%)	21 (10.4%)	
		More than 100,000	7 (6.5%)	70 (13.4%)	26 (8.8%)	3 (1.5%)	
		Don’t want to disclose	26 (24.1%)	132 (25.3%)	73 (24.6%)	91 (45.3%)	
	Occupation	Health workers	32 (27.8%)	215 (40.6%)	84 (27.6%)	19 (9.2%)	<0.001
		Teacher	18 (15.7%)	57 (10.8%)	33 (10.9%)	23 (11.1%)	
		Labourer	2 (1.7%)	14 (2.6%)	11 (3.6%)	12 (5.8%)	
		Unemployed	10 (8.7%)	29 (5.5%)	27 (8.9%)	25 (21.1%)	
		Private job	14 (12.2%)	52 (9.8%)	43 (14.1%)	20 (9.7%)	
		Own business	4 (3.5%)	29 (5.5%)	22 (7.2%)	9 (4.3%)	
		Retired	31 (27%)	121 (22.8%)	79 (26%)	92 (44.4%)	
		Others	4 (3.5%)	13 (2.5%)	5 (1.6%)	7 (3.4%)	
	Location	Khyber Pakhtunkhwa	64 (55.7%)	306 (57.7%)	184 (60.5%)	93 (44.9%)	0.007
		Punjab	23 (20%)	94 (17.7%)	56 (18.4%)	50 (24.2%)	
		Sindh	6 (5.2%)	31 (5.8%)	27 (8.9%)	14 (6.8%)	
		Balochistan	6 (5.2%)	18 (3.4%)	8 (2.6%)	16 (7.7%)	
		Federal / Islamabad	7 (6.1%)	35 (6.6%)	14 (4.6%)	15 (7.2%)	
		GB	3 (2.6%)	32 (6%)	14 (4.6%)	13 (6.3%)	
		AJK	6 (5.2%)	14 (2.6%)	1 (0.3%)	6 (2.9%)	
COVID-19 test and comorbid conditions	Have you ever tested positive for COVID-19	No	74 (64.3%)	350 (66%)	230 (75.7%)	154 (74.4%)	0.007
		Yes	41 (35.7%)	180 (34%)	74 (24.3%)	53 (25.6%)	
	Do you have an existing chronic disease such as diabetes, hypertension (blood pressure) or chest diseases (asthma etc)	No	100 (87%)	441 (83.2%)	270 (88.8%)	174 (84.1%)	0.150
		Yes	15 (13%)	89 (16.8%)	34 (11.2%)	33 (15.9%)	
COVID-19 Confidence	Rate your confidence in using UK, USA manufactured (imported) COVID-19 vaccine?	Completely confident	5 (4.3%)	150 (28.3%)	14 (4.6%)	9 (4.3%)	<0.001
		Confident	18 (15.7%)	243 (45.8%)	73 (24%)	44 (21.3%)	
		Neutral	29 (25.2%)	111 (20.9%)	166 (54.6%)	94 (45.4%)	
		Not Confident	34 (29.6%)	19 (3.6%)	41 (13.5%)	43 (20.8%)	
		Completely not Confident	29 (25.2%)	7 (1.3%)	10 (3.3)	17 (8.2%)	
	in using China manufactured COVID-19 vaccine?	Completely confident	3 (2.6%)	105 (19.8%)	8 (2.6%)	2 (1%)	<0.001
		Confident	17 (14.8%)	267 (50.4%)	67 (22%)	50 (24.2%)	
		Neutral	37 (32.2%)	124 (23.4%)	167 (54.9%)	100 (48.3%)	
		Not Confident	33 (28.7%)	21 (4%)	52 (17.1%)	38 (18.4%)	
		Completely not Confident	25 (21.7%)	13 (2.5%)	10 (3.3%)	17 (8.2%)	
	Vaccination decreases my chance of getting COVID-19 or its complications?	Strongly agree	2 (1.7%)	144 (27.2%)	17 (5.6%)	3 (1.4%)	<0.001
		Agree	23 (20%)	323 (60.9%)	133 (43.8%)	82 (39.6%)	
		Neutral	53 (46.1%)	56 (10.6%)	131 (43.1%)	102 (49.3%)	
		Disagree	23 (20%)	5 (0.9%	21 (6.9%)	18 (8.7%)	
		Strongly disagree	14 (12.12%)	2 (0.4%)	2 (0.7%)	2 (1%)	
	I am worried that the possible side-effects of COVID-19 vaccination would interfere with my routine activities?	Strongly agree	21 (18.3%)	70 (13.2%)	38 (12.5%)	19 (9.2%)	<0.001
		Agree	38 (33%)	206 (38.9%)	142 (46.7%)	106 (51.2%)	
		Neutral	43 (37.4%)	150 (28.3%)	99 (32.6%)	66 (31.9%)	
		Disagree	11 (9.6%)	87 (14.4%)	24 (7.9%)	13 (6.9%)	
		Strongly disagree	2 (1.7%)	17 (3.2%)	1 (0.3)	3 (1.4%)	
	I am concerned about the safety of the COVID-19 vaccination	Strongly agree	42 (36.5%)	154 (29.1%)	103 (33.9%)	75 (36.2%)	<0.001
		Agree	34 (29.6%)	165 (31.1%)	135 (44.4%)	94 (45.4%)	
		Neutral	33 (28.7%)	125 (23.6%)	56 (18.4%)	24 (11.6%)	
		Disagree	6 (5.2%)	74 (14%)	9 (3%)	11 (5.3%)	
		Strongly disagree	0 (0%)	12 (2.3%)	1 (0.3%)	3 (1.4%)	
	I am concerned about permissibility of COVID-19 vaccine on religious grounds?	Strongly agree	17 (14.8%)	38 (7.2%)	20 (6.6%)	16 (7.7%)	<0.001
		Agree	18 (15.7%)	92 (17.4%)	43 (14.1%)	24 (11.6%)	
		Neutral	54 (47%)	165 (31.1%)	137 (45.1%)	103 (49.8%)	
		Disagree	14 (12.2%)	134 (25.3%)	70 ()23%	46 (22.2%)	
		Strongly disagree	12 (10.4%)	101 (19.1%)	34 (11.2%)	18 (8.7%)	

## DISCUSSION

Out study highlights the issue of COVID-19 vaccine hesitancy. Less than half of the respondents would take COVID-19 vaccine. Confidence in using a UK or USA vaccine was more than a Chinese vaccine. This is very important in our context as the Chinese vaccines (Sinovac, Sinopharm and CanSino) are the mainstay of Pakistan COVID-19 vaccination drive.

Percentage of people who would not take the vaccine were comparable to the percentage of people who were concerned about the possible side effects of COVID-19 vaccination. Among the vaccine accepting group more than two thirds were male and less than one third were female.

Among the group who would decline to take the vaccine more than half were worried about the side effects. In the same group two thirds were concerned about the safety of the vaccine. Similarly, in the same group slightly less than one third were concerned about the religious permissibility of the COVID-19 vaccine. Two systematic reviews (worldwide) have reported that, vaccine acceptance was around 70%, and 61%.^13^,^14^ The highest acceptance was found in Ecuador (97%) and lowest in Kuwait (23%). Our study results lie in the middle of this wide range.

Looking at the regional vaccine acceptance, one global and another national study reported the vaccine acceptance from India to be 74%, China 89% and Saudi Arabia 65%).^15^,^16^ Acceptance in our study is way lower than this. The same global study reported the lowest acceptance from Russia (54%) but still higher than our results.^15^ Both the systematic reviews reported that acceptance was higher in Southeast Asia, males and health workers.^13, 14^ For male population our results are in line with this.

Regionally, India has reported 63% to 80% acceptance.^17^,^18^ In Bangladesh the vaccine acceptance ranged from 61% to 79%.^19^,^20^ In both the countries the acceptance has increased over time. Four studies from Pakistan reported the vaccine acceptance at 62%, 70%, 71 and 72%.^21^-^24^ Our study was conducted in January 2021while the other studies mentioned were conducted later that year. This shows that with time vaccine acceptance have increased in Pakistani population.

Another study reported vaccine acceptance in cardiac patients to be 49%.^25^

Our study findings suggest that amongst highly educated the vaccine acceptance is higher. In India one nationwide study reported differently, where with higher education the vaccine acceptance was lower.^26^ In a study from Bangladesh, no education and highest education had exactly the same vaccine acceptance with in between education levels showing higher vaccine acceptance.^27^ Though considering the different patterns of COVID-19 waves in these countries and the different timelines of availability of vaccines in these countries it is difficult to compare the patterns of vaccine hesitancy or acceptance. From Pakistan, Salman et al reported that the COVID-19 vaccination was highest (48%) among graduates. It was remarkably low in all other education level. Another study from Pakistan reported that the higher the education the higher the vaccine acceptance.^28^ Both these studies results are in line with our findings. Our study results are important for the policy makers and all the relevant stakeholders who are implementing or assisting in implementation of the vaccination campaigns in Pakistan about the low vaccine acceptance and its associated factors.

### Limitations

One limitation of our study is that this was an online survey and the people who participated were the ones with access to internet or social media.

## CONCLUSION

COVID-19 vaccine hesitancy exists in Pakistan. Gender and marital status were significantly associated with vaccine acceptance. The respondents who were vaccine hesitant, had concerns over its side effects, safety and religious permissibility of vaccine. This could be a major bottle neck in controlling the COVID-19 pandemic despite the availability of the vaccines in Pakistan.

### Recommendations:

As vaccines become more readily available throughout Pakistan, focus should be on planning a good communication plan. The plan should focus on all the relevant factors to increase vaccine acceptance.

### Supplementary materials:

Table-I and Table-II are available online, in supplementary materials.

### Informed consent statement:

Informed consent was obtained from all subjects involved in the study.

### Authors’ contributions:

**KR:** Contributed in conceptualization, methodology, reviewing and editing the draft,

Supervised the process. He is responsible for the accuracy and integrity of this study.

**NA:** Contributed in formal analysis, data curation, writing and preparation of original draft.

**MJ:** Contributed in analysis, writing and preparation of original draft, reviewing and editing the draft.

**AM:** Contributed in methodology, data collection, writing and preparation of original draft.

**IK:** Contributed in methodology, data collection, data curation, writing and preparation of original draft.

**MA:** Contributed in conceptualization, methodology, reviewing and editing the final draft.
